# Progress in the Synthesis of Bifunctionalized Polyhedral Oligomeric Silsesquioxane

**DOI:** 10.3390/polym11122098

**Published:** 2019-12-14

**Authors:** Mingyue Wang, Hong Chi, Joshy K.S., Fuke Wang

**Affiliations:** 1 Shandong Provincial Key Laboratory of Molecular Engineering, School of Chemistry and Pharmaceutical Engineering, Qilu University of Technology (Shandong Academy of Sciences), Jinan 250353, China; Silvia9607@163.com (M.W.);; 2 Institute of Materials Research and Engineering, A*STAR (Agency for Science, Technology and Research), Fusionopolis Way, Innovis, #08-03, Singapore 138634, Singapore

**Keywords:** polymer composite, bifunctional POSS, asymmetry, functionalization

## Abstract

Polyhedral oligomeric silsesquioxane (POSS) has been considered as one of the most promising nanofillers in academic and industrial research due to its unique multifunctional nanostructure, easy functionalization, hybrid nature, and high processability. The progress of POSS has been extensive, particularly applications based on single- or multiple-armed POSS. In polymer hybrids, in order to enhance the properties, bifunctional POSS has been incorporated into the backbone chain of the polymer. This review summarizes recent developments in the synthesis, modification, and application of bifunctional POSS-containing composite materials. This includes amino-POSS, hydroxyl-POSS, aromatic ring-POSS, ether-POSS, and vinyl groups-POSS and their applications, exemplified by polyurethanes (PUs) and polyimides (PIs). In addition, the review highlights the enhancement of thermal, mechanical, and optical properties of the composites.

## 1. Introduction

In recent years, inorganic–organic hybrid materials with carbon nanotubes [1,2,3,4,5], graphene [6,7,8,9], organosilicons [10,11,12], metal oxides [13,14], and natural polymers [15,16,17] have attracted considerable attention because of their distinctive properties. Among these, the incorporation of polyhedral oligomeric silsesquioxane (POSS) has attracted attention because of its unique frame structure and the resulting chemical and physical properties, including high thermal properties [18,19,20], lower crystallinity [21], mechanical properties [22], oxidation resistance, and very good dielectric properties [23,24]. Hence, it has been widely used in preparing polymer hybrids and polymer composites in many areas, including optics [25], electronics, ionic liquids [26], mechanics [27], energy [28], environment [29], biology [30], smart coatings [31,32], fuel [33], solar cells [34], catalysts [35,36], sensors [37,38,39], light-emitting devices [40], and medicine [41,42,43,44].

With a silicon core and surrounding organic functional groups, POSS itself is a hybrid material at the molecular level. It is composed of a cubic polyhedron cage surrounded by multiple silicon oxygen rings. The substituents on the Si atom at the vertex of the POSS polyhedron can be a variety of reactive or nonreactive groups. The desired properties can be obtained by alternating the types of organic groups on the Si endpoint that determines whether POSS is reactive or functionalized [45,46]. POSS enhances the mechanical properties of polymers (e.g., modulus, strength, hardness) and decreases heat evolution and melt viscosity [17,47]. POSS increased the chain rigidity of polymers, and as a result, the glass transition temperatures (*T*_g_) were greatly enhanced. High-molecular-weight copolymers will be obtained by incorporating POSS moieties, which will result in improved mechanical properties. Due to this excellent property, these polymeric materials have found wide application in both academic and industrial fields. For example, hybrid POSS/polyurethane (PU) material has been applied for the construction of synthetic heart valve leaflets [48] and aortic stent grafts in medicine [49].

The classic eight-armed POSS-containing hybrids normally form cross-linked networks, hence they exhibit poor solubility in most organic solvents. On the other hand, single-armed POSS macromeres used as pendent [50,51,52] or cross-linking sites will not affect the main chains or overall properties of POSS-containing polymers. Bifunctional POSSs (B-POSSs) are emerging silsesquioxanes that are usually incorporated into the backbone of polymers, leading to the occurrence of several new functions, such as nonflammability, oxidative resistance, and excellent dielectric properties [53,54,55,56]. When B-POSSs are introduced into the main chain of a polymer, the improved performance of the polymer is mainly due to the altered motion of the polymer chains. Thus, B-POSS has received attention because of its fascinating properties, such as good thermal and mechanical properties, excellent transparency, and excellent flexibility [57,58,59]. For example, Wei et al. [60] successfully synthesized a B-POSS through a click reaction between 3,13-diazidopropyloctaphenyl double-decker silsesquioxane (DDSQ; compound **18**) and *α*,*ω*-dialkynyl-terminated oligoethylenes. This B-POSS showed high thermal stability, high hydrophobicity, and low surface energy. It also showed many excellent properties, which makes it a promising candidate for flexible substrates and polymer electronics. For example, B-POSS itself is a hydrophobic material; thus, it is not inclined to adhere to metals. Mohammod et al. [55] reported the surface modification of B-POSS by the incorporation of silanol groups (Si–OH) in the film. The surface of B-POSS film changed from hydrophobic to hydrophilic by irradiation with deep ultraviolet (UV) radiation. The deep UV radiation cleaved the Si–O–Si cage of silicones. Silver (Ag) micropatterns on the surface of B-POSS film were also fabricated by the deep UV irradiation process. The obtained Ag micropatterns exhibited excellent adhesion on the modified surface.

The composites usually show many interesting properties, such as amphiphilic [61], thermal [62], and optic properties [63], and also form complexes using coordination chemistry [64]. The incorporation of B-POSS into the main chains of a polymer results in significant lowering of the dielectric constant. This is because B-POSS has an inert inorganic silicon framework [65]. In addition, B-POSS-based materials possess desirable chemical and physical properties due to their structural and property variability, which is tuned by the modulation of functional groups. Sodkhomkhum et al. [66] reported the synthesis of poly(siloxane/B-POSS) via a polycondensation reaction between chlorides containing B-POSS and hexamethyltrisiloxane with high *T*_g_ and transparency. *Iso*-butyl end-capped B-POSS reported by Groch et al. [67] showed enhanced solubility compared with methyl end-capped B-POSS. Thermal properties investigated by thermogravimetric analysis (TGA) showed that the polymers had exceptionally high thermal degradation temperature (*T*_d_) in the range of 470–530 °C whether in nitrogen or air atmosphere. About 80% residual weight of the polymers was found in nitrogen atmosphere at 760 °C.

Due to the promising applications of B-POSS, this review focuses on its synthesis and functionalization methods. The applications and properties of polymers containing POSS will also be introduced. Finally, we suggest some prospects for POSS from our own perspective.

## 2. Functional Methods of B-POSS

**Figure 1:**
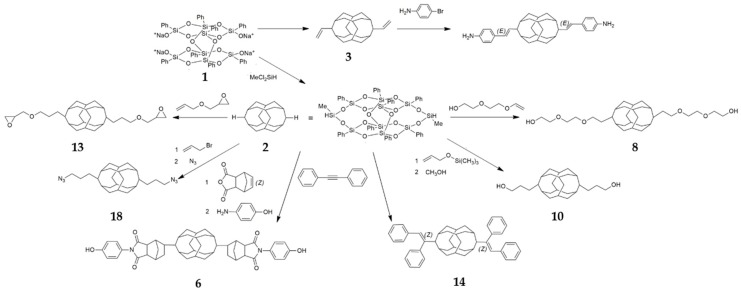
Schematic illustration of reaction types of bifunctional polyhedral oligomeric silsesquioxanes (B-POSSs) including octaphenyldicycloocatasiloxane tetrasodium Silanolate (**1**), 3,13-dihydrooctaphenyl B-POSS (**2**), 3,13-divinyl B-POSS (**3**), para-aminophenol anhydride B-POSS (**6**), di(ethylene glycol) POSS (**8**), 3,13-Dihydroxypropyloctaphenyl B-POSS (**10**), 3,13-diglycidyloxypropyloctaphenyl B-POSS (**13**), 3,13-diphenylethenyl B-POSS (**14**), 3,13-diazidopropyloctaphenyl B-POSS (**18**). Numbers denote compounds discussed in the paper.

Figure 1 shows the schemes of various synthetic possibilities of B-POSS. Generally, B-POSS is synthesized through reactions between raw materials such as phenyltrimethoxysilan, isopropanol, sodium hydroxide, and methylvinyl dichlorosilane or methyldichlorosilane. B-POSS has been obtained by involving the synthesis of 3,13-dihydrooctaphenyl B-POSS (**2**) and bifunctional POSS. During the synthesis of B-POSS, Karstedt catalyst was used and tetrahydrofuran (THF) toluene or isopropanol were employed as solvent. The obtained B-POSS was washed with methanol or hexane and characterized by ^1^H-NMR, ^29^Si-NMR, (Fourier transform infrared spectroscopy) (FT-IR ) analysis.

### 2.1. Amino-Functionalized POSS

**Figure 2:**
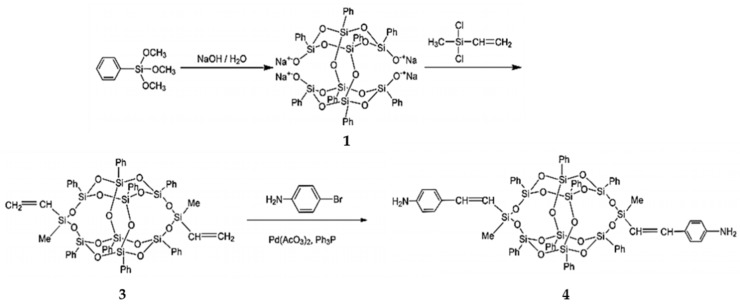
Synthesis of 3,13-dianilino B-POSS (**4**). (Reprinted with permission from Liu et al. [68]. Copyright 2016 Royal Society of Chemistry).

Liu et al. synthesized diamine-modified POSS (**4**) by means of a Heck reaction, as shown in Figure 2 [68]. An ocatasiloxane tetrasodium silanolate (denoted Na_4_O_14_Si_8_(C_6_H_5_)_8_) (compound **1**) was first obtained through the hydrolysis of a phenyltrimethoxysilane precusor in isopropanol by using the same procedure as Kakimoto et al. [69]. Phenyltrimethoxysilane, isopropanol, and sodium hydroxide were used as the precursor, solvent, and catalyst, respectively. Diamine-modified POSS was obtained through the reaction between 3,13-divinyl B-POSS (**3**) and 4-bromoaniline with palladium catalyst (Figure 2), with a yield of 91%. The structures of compounds **3** and **4** were verified by ^1^H-NMR. Compared with one-armed POSS, compound **4** can be adopted to synthesize polymer hybrids containing B-POSS in the backbone, including polyamide (PA), polyurethane (PU), and polyimide (PI), through step polymerization, where the activity of the amine functional group is of prime concern.

**Figure 3:**
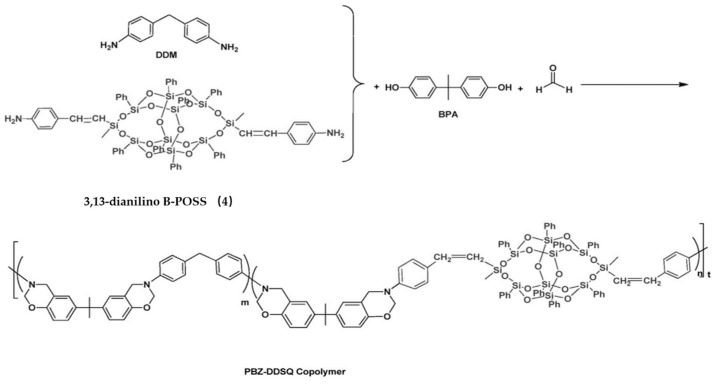
Synthesis of organic–inorganic polybenzoxazine-B-POSS (PBZ-B-POSS) copolymers based on compound **4**. (Reprinted with permission from Liu et al. [70]. Copyright 2017 Elsevier).

Based on compound **4**, Liu et al. [70] prepared polybenzoxazine-B-POSS (PBZ-B-POSS) copolymers, as shown in Figure 3. For this, compound **4** was introduced into main chains of polybenzoxazines (PBZs) by reaction with 4,4-diaminodiphenylmethane (DDM), 4,4′-dihydroxyldiphenylisopropane, and formaldehyde. Compared to neat PBZ, the hybrid copolymers exhibited enhanced surface hydrophobicity with increased content of B-POSS. The contact angle of PBZ-B-POSS copolymers was significantly higher than that of neat PBZ (99.6°). PBZ-B-POSS thermoset copolymers can be easily obtained by thermally activated ring-opening polymerization.

Based on compound **2**, Wu et al. [71] reported the synthesis of aromatic tetracarboxylic dianhydride modified B-POSS via the reaction between 3,13-dianilino B-POSS (**4**) and pyromellitic dianhydride. The hybrid exhibited enhanced solubility, 5% weight loss (*T*_5%_) at 570 °C in nitrogen atmosphere, and a high *T*_g_ of 300 °C. On the other hand, tetracarboxylic dianhydride modified B-POSS can be synthesized before the modification of diamine groups, as exemplified by Wu et al. [72]. Double-decker-shaped silsesquioxane dianhydride was synthesized before further reaction with 4,4-oxydianiline. ^29^Si-NMR confirmed their structure by the appearance of peaks at intensity values of −21.8, −78.3, and −78.9 ppm.

**Figure 4:**
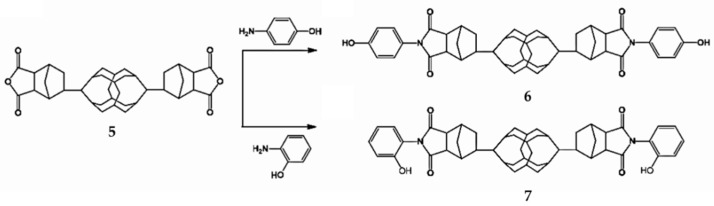
Synthesis of compounds B-POSS-ND-*p*-OH (**6**) and B-POSS-ND-*o*-OH (**7**) from **5**. (Reprinted with permission from Chen et al. [73]. Copyright 2018 American Chemical Society).

When 4,4-oxydianiline is replaced by p-aminophenol or o-aminophenol, hydroxyl group modified B-POSS can be formed, as reported by Chen et al. (Figure 4). [73] First, compound **5** was reacted with p-aminophenol or o-aminophenol to form the para-aminophenol anhydride B-POSS (B-POSS-ND-*p*-OH) (**6**) and ortho-aminophenol anhydride B-POSS (B-POSS-ND-*o*-OH) (**7**), as shown in Figure 4. The influence of the substitution position of the B-POSS on the thermal stability and thermal behavior of these (benzoxazine) (BZ) monomers was well investigated. The thermal investigation of B-POSS-BZ monomers proved that the incorporation of B-POSS enhanced their resistance to the degradation of the hybrid monomers. The ortho-substitution of the aminophenol units resulted in lower thermal stability than the para-substitution of the aminophenol units.

### 2.2. Hydroxyl Functionalized POSS

**Figure 5:**
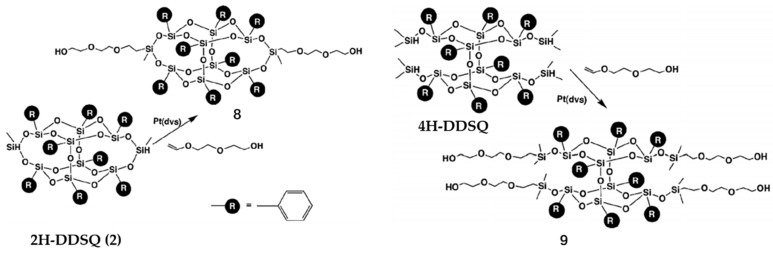
Synthesis of 4OH-B-POSS (**9**) of four hydrogen double-decker silsesquioxane (4H-DDSQ) with di(ethylene glycol) vinyl ether via hydrosilylation. (Reproduced with permission from Kucuk et al. [76]. Copyright 2011 Elsevier).

Due to the strong aggregation tendency of the super-hydrophobic POSS moiety [74], we reviewed the mechanism of amphiphilic hybrid POSS copolymer in self-assembly, in which POSS can effectively control the motion of the polymer chain [75]. Kucuk et al. reported the preparation of the first (Langmuir-Blodgett film) (L-B film) containing B-POSS with a well-defined structure [76]. Amphiphilic two- and four-armed di(ethylene glycol) POSS (2OH-B-POSS) (**8**) and 4OH-B-POSS (**9**) were synthesized. 2OH-B-POSS was formed by the reaction between 2H-B-POSS and a 1.3-fold excess amount of di(ethylene glycol) vinyl ether by means of a direct hydrosilylation reaction (Figure 5). Similarly, 4OH-B-POSS was obtained, and the structure was confirmed by ^1^H-NMR. Through surface pressure-area (π-A) isotherms and Brewster angle microscopy, the amphiphilic B-POSS monolayer at the air–water interface was observed.

To further probe the hydrogen bonding effect on the monolayer properties, they also synthesized amphiphilic B-POSS with core-corona amphiphile full name 2-di(ethylene glycol) urethane B-POSS (2DEGNH-B-POSS) [77]. Ethyl isocyanate and 2-di(ethylene glycol) B-POSS (2DEG-B-POSS) were used as raw materials to fabricate 2DEGNH-B-POSS. The structure was confirmed via ^1^H-NMR and FT-IR. The 2DEGNH-B-POSS with a urethane group at the end exhibited a strong hydrogen bond interaction in the 2DEGNH-B-POSS rod-like assemblies. A uniform liquid-like monolayer was formed by the mixture of 2DEGNH-B-POSS and full name 2DEG-B-POSS (1:1 molar ratio). Monolayer properties could be improved because of the hydrogen bonds between the urethane groups in 2DEGNH-B-POSS and the hydroxyl groups in 2DEG-B-POSS, which guaranteed the successful separation of L-B film.

**Figure 6:**
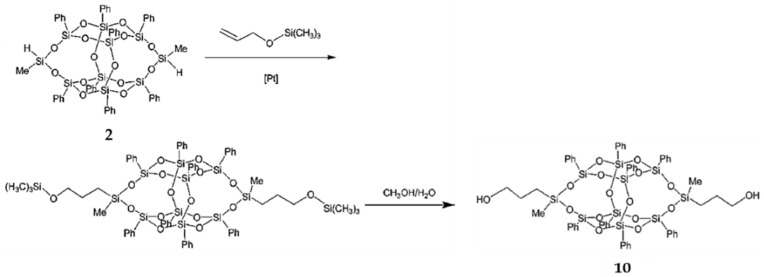
Synthesis of 3,13-dihydroxylpropyloctaphenyl B-POSS (**10**). (Reproduced with permission from Wei et al. [78]. Copyright 2012, Royal Society of Chemistry).

3,13-Dihydroxypropyloctaphenyl B-POSS (**10**) was synthesized by Wei et al. [78]. First, 3,13-di(trimethylsilyl)oxypropyloctaphenyl B-POSS was obtained by means of a hydrosilylation reaction between B-POSS and allyloxytrimethylsilane. Then, compound **11** was obtained through a deprotection reaction of 3,13-di(trimethylsilyl)oxypropyloctaphenyl B-POSS (Figure 6). ^1^H-NMR confirmed their structure by the appearance of a CH_3_–Si group at 0.38 ppm, Si–H group at 4.98 ppm, benzene rings at 7.14–7.50 ppm, and –OSiCH_3_CH_2_CH_2_CH_2_OH group at 0.31 ppm. Then, compound **10** was used as a chain extender to produce linear hybrid PUs with B-POSS in the backbone. These organic–inorganic linear PUs showed enhanced surface hydrophobicity. It was also noted that the *T*_g_ values increased with the increased content of B-POSS.

Wen et al. [79] synthesized epoxy soybean oil-based polyurethanes modified by 3,13-dimethyhydroxysilyl double-decker phenylsilsesquioxane (B-POSS(Me)OH). B-POSS(Me)OH was directly obtained by the reaction between compound **1** and methyltrichlorosilane. ^1^HNMR and MALDI–TOF–MS confirmed the successful synthesis of B-POSS(Me)OH. TGA was applied to investigate the thermal properties of hybrid polyurethanes. At high temperature, the thermal stability and flame resistance of the hybrids were significantly improved because of the silicon dioxide generated from the oxidation of B-POSS(Me)OH wrapping on the surface. Differential scanning calorimetry (DSC) showed that the *T*_g_ of the hybrid polyurethane exhibited a parabolic linear relation with the increased B-POSS(Me)OH content. By incorporating different contents of B-POSS(Me)OH, the mechanical property of hybrid polyurethane varied, and the amount exceeded 4.6 wt %, and the tensile strength started to decrease.

**Figure 7:**
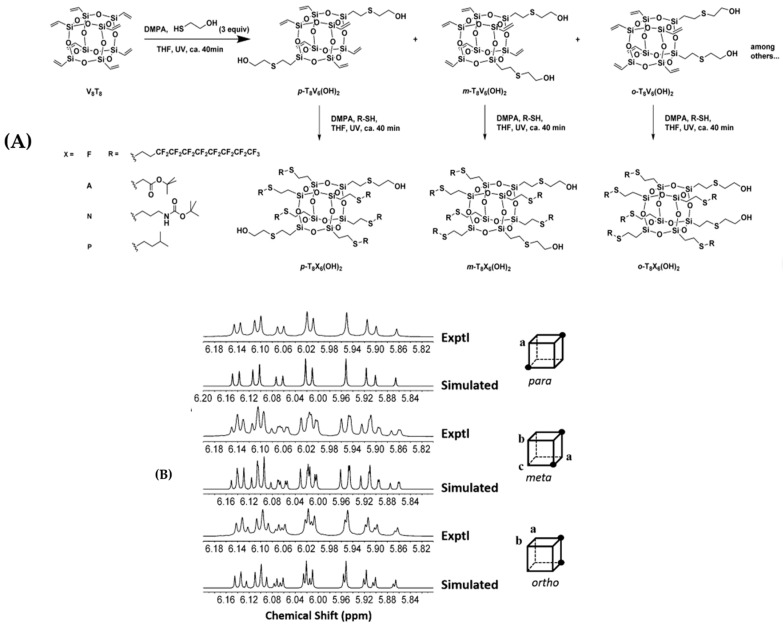
(**A**) Synthesis of para-, meta-, and ortho-T_8_X_6_(OH)_2_ (X = V, F, A, N, or P) with hydroxyl groups. (**B**) Experimental and simulated ^1^H NMR spectra of the products. Letters indicate chemically inequivalent vinyl groups on the POSS cage. (Reprinted with permission from Han et al. [80]. Copyright 2016 John Wiley and Sons).

Han et al. [80] reported a variety of Janus-type regioisomeric B-POSSs functionalized by hydroxyl groups, which were synthesized through thiol–ene “click” chemistry (Figure 7). ^1^H NMR and ^13^C NMR were successfully applied to differentiate and study a class of mixed octakis-adduct regioisomers with various surface positional functional groups. Due to the existance of equivalent vinyl groups, the para-substitutional product *p*-T_8_V_6_(OH)_2_ only exhibited one set of strongly coupled, second-order spectra of an ABC spin system by ^1^H NMR. Tiny differences in the chemical shifts beween experimental and simulated spectra were found in *m*- and *o*-T_8_V_6_(OH)_2_ (meta or ortho-substitutional product which obtained by the V_8_T_8_ showed in the Figure 7). The spectral pattern presented a group of signals from each individual subset of vinyl resonance signals. The ^13^C NMR spectra of *p*-, *m*-, and *o*- T_8_V_6_(OH)_2_ were also classified according to the differences between them.

### 2.3. Aromatic Ring Functionalized POSS

**Figure 8:**
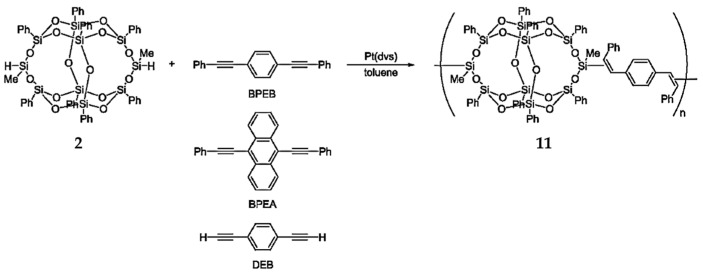
Hydrosilylation polymerization of B-POSS with diynes. (Reproduced with permission from Seino et al. [69]. Copyright 2006 American Chemical Society).

To synthesize high-performance polymers, POSSs are often functionalized with aromatic groups [81,82,83,84]. Seino et al. [69] reported that the reaction between B-POSS and diynes resulted in the formation of linear polymers via hydrosilylation polymerization. Compound **11** was formed quantitatively through the reaction of compound **2** with diphenylacetylene (Figure 8). The polymerization of B-POSS with diynes was obtained via hydrosilylation, as shown in Figure 8. Chemical structures were confirmed by ^1^H-NMR spectroscopy; the characteristic peaks of B-POSS were methyl groups ranging from 0.22 to 0.38 ppm, and the characteristic peaks of the phenylene group signals appeared from 6.41 to 7.59 ppm.

Benzocyclobutene (BCB) is a moiety that can be introduced into polymers and oligomers due to its high refractive index, stable air and moisture exposure, low dielectric constant, and excellent thermal and mechanical properties. A series of benzocyclobutene-functional B-POSSs (2BCB-B-POSS and 4BCB-B-POSS) were synthesized by Hu et al. [85]. After curing at above 200 °C, the hybrid resins were converted into highly cross-linked polymers with enhanced thermal stability and optical and electrical properties via Diels–Alder reaction. Compared with resin cured without B-POSS, this BCB-B-POSS showed better performance in terms of low water abosorption, low heat transfer, low dielectric constant, and higher (light emitting diode) (LED) luminous efficiency. A new type of bifunctional phenolic B-POSS (B-POSS-BP) was synthesized with allylamine and CH_2_O via Mannich condensation to form a bis-allyl benzoxazine B-POSS derivative (B-POSS-BZ) by Liao et al. [86]. The B-POSS was critical in preparing thermally stable, optically transparent, and mechanically flexible polybenzoxazine polymers after the hydrosilylation of B-POSS-BZ with polydimethylsiloxane (PDMS).

### 2.4. Divinyl Functionalized B-POSS

**Figure 9:**
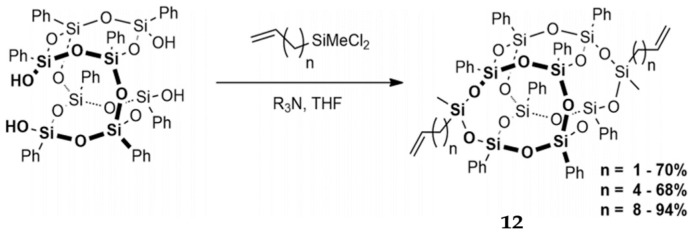
Synthesis of dialkenylfunctional B-POSS (**12**) via condensation reaction. (Reprinted with permission from Mituła et al. [87]. Copyright 2017 Creative Commons CC BY license).

From the synthetic point of view, divinyl substituted B-POSS is important in free radical polymerization and silylative coupling. Mituła et al. synthesized dialkenyl-substituted B-POSS, including allyl, hex-5-enyl-, and dec-9-enyl-B-POSS, by condensation and hydrosilylation processes (Figure 9) [87]. First, dichloro(alkenyl)methylsilane was obtained by the reaction between dichloromethylsilane and 1,9-decadiene under the [Pt_2_(dvds)_3_] catalyst. Then, B-POSS-2Si-decenyl was obtained by the condensation reaction between B-POSS-4OH and dichloro(dec-9-enyl)methylsilane under triethylamine (R_3_N) and THF. B-POSS-2-Si-Allyl (*n* = 1), B-POSS-2-Si-hexenyl (*n* = 4), and B-POSS-OH (*n* = 8) were also obtained by the same reaction. Walczak et al. [88] also reported the synthesis of a series of B-POSS functionalized highly π-conjugated ethylarenes and analogous hybrid materials. The existance of compound **2** with both cis- and trans-isomers was verified by ^29^Si NMR. The appearance of 18.35, 78.51, and 79.46 ppm peaks and 18.35, 78.51, 79.40, and 79.49 ppm peaks was attributed to the trans- and cis-isomers, respectively. This new B-POSS compound was reacted with styryl- and bis(styryl)arenes via hydrosilylation reaction, leading to the formation of ethyl bridged molecular and macromolecular hybrid systems.

Along with silylative coupling, cross-metathesis provides efficient and complementary synthetic routes, leading to B-POSS hybrids of great practical importance. Zak et al. [89] produced a series of functionalized dialkenyl silsesquioxanes. Two types of transititon metal-catalyzed reactions, highly stereoselective silylative coupling (SC) and cross-metathesis (CM), of divinyl substituted B-POSS (B-POSS-2SiVi) with substituted styrenes and other olefins were used for the synthesis, resulting in the formation of E isomer quantitatively.

After that, silylative coupling and metathetic copolymerization (ADMET) were further used to synthesize a new class of vinylene-arylene and B-POSS copolymers in another work [90]. Different arenes were polymerized in order to compare their thermal and mechanical properties. TGA proved high thermal resistance over 550 °C, depending on the B-POSS content. Gel permeation chromatography (GPC) measurements confirmed that the average molecular weight (*M*_w_) of copolymers improved with the elongation of reaction time, which was possibly due to the steric hindrance.

It was found that the activity of the catalyst was affected by the feeding ratio of B-POSS in the copolymerization. Groch et al. [91] synthesized copolymers of ethylene with divinyl substituted B-POSS by coordinative copolymerization under the catalyst of metallocene and bis(phenoxy-imine). The B-POSS content varied in the range of 0.93–11.53 wt % to optimize and obtain the relation between compositions and the structural properties of copolymers. Different products were obtained depending on the different B-POSS content, pressure of ethylene, and time of reaction. Results indicated that with increased feeding concentration of B-POSS, the activity of metallocene catalyst was found to initially increase and then gradually decrease over a critical value.

### 2.5. Asymmetric Functionalization of B-POSS

Incorporating B-POSS in the polymer chain provides a more effective retardation of the chain motion of the linear polymer, which might allow higher effectiveness in property modulation. However, it is noteworthy that B-POSS bridging different segments or chains on each side may offer an interesting new class of materials.

**Figure 10:**
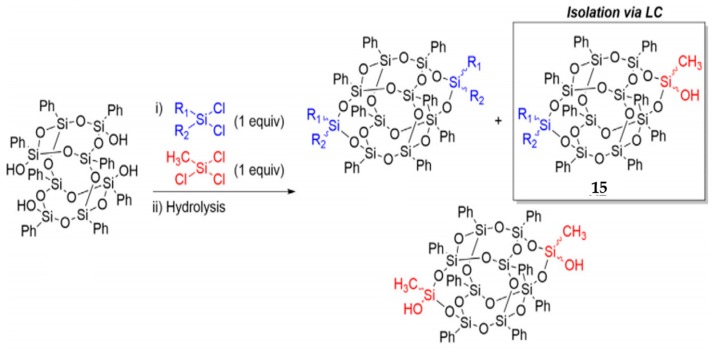
Asymmetric side-capped B-POSS by using a combination of dichloro- and trichlorosilane capping agents. (Reprinted with permission from Vogelsang et al. [92]. Copyright 2018 Elsevier).

Vogelsang et al. [92] reported a method to obtain asymmetric side-capped B-POSS by using a combination of dichloro- and trichlorosilane capping agents (Figure 10). A mixture of symmetric and asymmetric B-POSSs was obtained by the reaction between B-POSS(OH)_4_ and (CH_3_)(R)SiCl_2_, (CH_3_)SiCl_3_, as shown in Figure 10. To obtain AB type, liquid chromatography (LC) was considered as an effective separation technique and was employed to obtain compound **15** (AB) with asymmetric functionality. However, significant symmetric byproduct waste would be generated and it required differences in polarity between the byproducts and the desired asymmetric material to be effectively separated.

**Figure 11:**
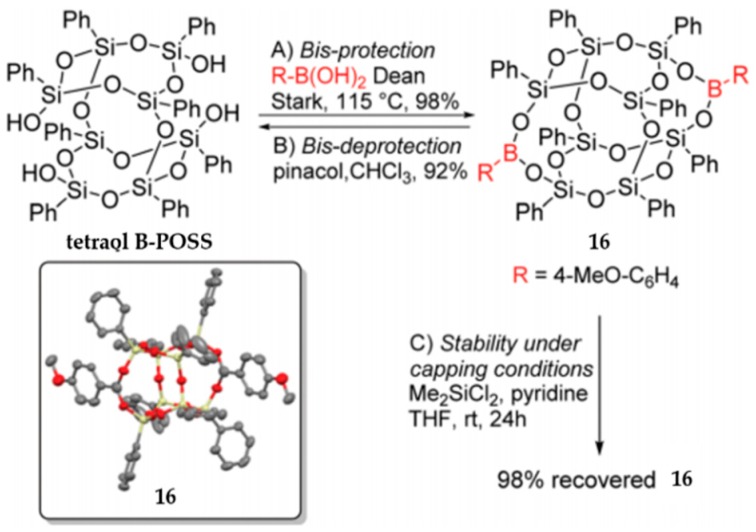
Asymmetric functional B-POSS by the selective protection of silanols with boronic acid. B represents the boron atom and the R on the boron atom represents 4-Me-C_6_H_4_, which are more clearly to show selective-protected location of the tetraol B-POSS. (Reprinted with permission from Barry et al. [93]. Copyright 2019 Royal Society of Chemistry).

Based on Vogelsang‘s work, Barry et al. reported a more effective route to synthesize asymmetric functional B-POSS (**16**) by the selective protection of silanols with boronic acid (Figure 11) [93]. Such an active protecting group could protect two silanols simultaneously, and it could be easily introduced and removed without affecting the B-POSS framework. In this way, high recyclable starting tetraol B-POSS was also achieved. 4-Methoxyphenylboronic acid was used because its outstanding methoxy protons gave a high yield (98%) and simple spectroscopic analysis was allowed. The protocol is general and highly efficient for a wide range of asymmetrically functionalized B-POSSs.

A special catalyst was also explored in the selective synthesis of asymmetric B-POSS. Zak et al. reported ruthenium-N-heterocyclic carbene complexes (formula [RuHCl(CO)(NHC)(PCy_3_)]) as efficient and selective catalysts [94]. The [RuHCl(CO)(NHC)(PCy_3_)] complexes were obtained by ligand exchange between tricyclohexylphosphine (PCy_3_) and N-heterocyclic carbene (NHC). The high catalytic activity exhibited by the new catalyst allowed the bifunctionalization of three divinyl-substituted B-POSS cage compounds in one pot. Tian et al. [95] synthesized a kind of asymmetric functional B-POSS with silyl hydride at one end and two silanol groups at the other end. Here, only one of the two silanol groups coupled with silyl hydride, while the other silanol remained intact owing to steric hindrance. Blanco et al. [96] prepared POSS/PS hybrids by in situ polymerization to compare the effect of symmetry and asymmetry POSSs in terms of thermal property. The asymmetry POSS/PS showed better thermal performance indicated by TG, (differential thermogravimetric) (DTG), and DSC. Tanaka et al. [97] also investigated the thermal and mechanical properties of a series of polymers such as polystyrene (PS), poly(methyl methacrylate) (PMMA), and ethylene-(vinyl acetate) copolymer (EVA), which were incorporated with 5 wt % POSS. The polymer matrices showed good thermal stability because of the incorporation. The SEM suggested the homogeneous dispersion of POSS. The POSS fillers were found to enhance the hardness of PS, which was confirmed by (dynamic mechanical analysis) (DMA). Hence, obtaining asymmetrical POSS molecules is highly important for dispersion in the polymer matrix.

### 2.6. Other Bifunctional POSS

**Figure 12:**
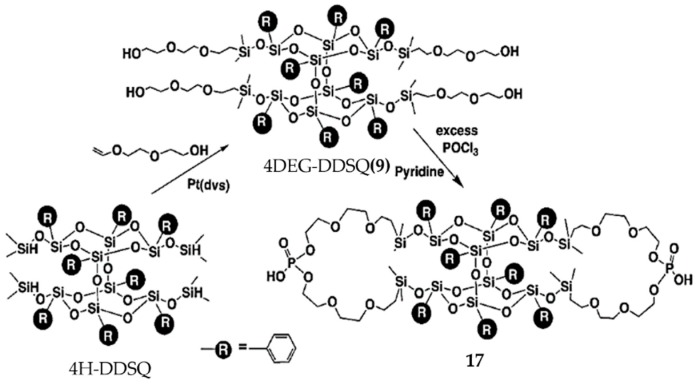
Synthesis of phosphonic-acid-containing B-POSS (PHOS-B-POSS) (**17**). (Reproduced with permission from Kucuk et al. [98]. Copyright 2012 Royal Society of Chemistry).

Chlorine-containing bifunctional POSS (Cl-B-POSS) with excellent optical transparency and hydrophobicity was obtained via the polycondensation reaction of di[(3-chloropropyl)isopropoxysilyl]-bridged B-POSS with hexamethyltrisiloxane by Sodkhomkhum et al. [66]. Phosphonic-acid-containing B-POSS (PHOS-B-POSS) (**17**) was synthesized by Kucuk et al. to prepare proton-conducting electrolyte film [98]. First, four-armed di(ethylene glycol) B-POSS [4DEG-B-POSS] (**9**) was obtained via hydrosilylation reaction using 4H-B-POSS and di(ethylene glycol) (DEG) vinyl ether, with Pt(dvs) as catalyst. Then, compound **17** was synthesized through the reaction between compound **9** and (phosphorus oxychloride) (POCl_3_) (Figure 12). ^1^H-NMR spectra suggested the appearance of –CH_2_–O–P at 3.66 ppm. DSC indicated that *T*_g_ was below room temperature. Compound **17** cast film exhibited high conductivity (0.12 S cm^–1^, 85 °C) under 95% relative humidity. Compound **17** showed its possible application in fuel cells because of this excellent proton conductivity under humid conditions as well as high thermal stability. Another phosphorus-containing B-POSS used as flame-retardant material was reported by Song et al. [99]. Novel silicon–phosphorus linear polymers were synthesized via hydrosilylation reaction between B-POSS and 9,10-dihydro-9-oxa-10-phosphaphenanthrene-10-oxide derivatives. Then, different contents of B-POSS were blended with polycarbonate/acrylonitric–butadiene–styrene (PC/ABS) to study the flame-retardant properties. The temperature of 5 wt % weight loss (*T*_5%_) and the char residue yield at 800 °C suggested good flame retardancy of PC/ABS containing B-POSS.

With a special substituted difluorosilane as a precursor, Tanaka et al. [100] reported a substituted difluorosilane prepared from the reaction between BF_3_·Et_2_O and siloxanolate in the synthesis of an amide product. The difluorosilane, bearing a vinyl- or BF_3_-complexed amino group, was used as the raw material to synthesize polycyclic silsesquioxanes under mild conditions. This general strategy enabled the synthesis of B-POSS where various functional groups could be introduced.

3,13-Diglycidyloxypropyloctaphenyl B-POSS (**13**) was synthesized by Wang et al. via a hydrosilylation reaction between 3,13-dihydrooctaphenyl B-POSS (**2**) and allyl glycidyl ether [101]. Based on compound **13**, Zhang et al. [102] further incorporated this B-POSS macromer into polybenzoxazine (PBZ) to investigate the thermal and mechanical properties through dynamic mechanical thermal analysis (DMTA) and TGA. The *T*_g_ value was higher than the PBZ and increased with the increased content of di-functional POSS macromere. With various amounts of 3,13-diglycidyl B-POSS, the nanocomposites containing 30 wt % of POSS showed the highest *T*_g_ value. The *T*_d_ increased with the increased compound **13** content.

Mono- and poly-epoxy groups containing B-POSS were synthesized by Cao et al. [103]. Curing kinetics and the thermal and mechanical performance of epoxy resin with varying amounts of B-POSS loading ratios were studied. The mono B-POSS showed more flexible structure and a better toughening effect, while the branched poly B-POSS exhibited higher thermal resistance.

A class of B-POSS-functionalized di-nuclear alkynylplatinum (II) terpyridine complexes exhibiting stereoisomerism was reported by Au-Yeung et al. [104]. The cis- and trans-complex stereoisomers were separated by column chromatography and identified by X-ray crystallography. The cis-isomer showed that the two methyl groups located on the silicon atoms pointing in the same direction exhibited a small C–Si–Si–C torsion angle of 13.71°, whereas the trans-configuration showed a torsion angle of 180°. The cis-isomer also exhibited significantly different aggregation behavior in 50% water–acetone mixture. In 60% water–acetone solution, the cis-form showed a morphological transformation into spheres with a diameter of about 90 nm.

## 3. Polymer Composite*s* Containing B-POSS

Linear polymers refer to molecular chains that are linear and irregular. Due to the simplicity of materials processing, good solubility, high chain regularity, and POSS content in the hybrids [89,105], more attention has been paid to B-POSS-containing linear composite materials because of the specific nanostructure, hydrophobic core, functionalization, flame retardance, optical transparency, and low thermal conductivity.

### 3.1. PU–POSS

In recent years, PU has been applied in various areas such as packaging, coatings, footwear, and consumer care products [106]. The applications have been extended to the biomedical field due to the biocompatibility of PUs [107]. Even the liquid crystalline PU (LCPU) field, when LCPU was incorporated into POSS, the LCPU/POSS hybrids could show high melting and isotropization temperatures along with the broadening phase transition effect due to the incorporation of POSS [108]. Although PUs have been used in many fields, they still have many limitations, such as poor thermal and moisture stability originating from urethane groups and low mechanical resistance due to the intrinsic hardness of the hard segments. Therefore, many efforts have been made to solve these problems. The incorporation of POSS could affect the microphase-separated morphology, dynamics of phase separation, and order development of PU with different annealing temperatures. At low temperature, the POSS could promote the phase separation; however, the phase separation would slow under the high temperature [109]. Structural and property modifications of linear PUs with POSS have attracted much attention [110,111,112].

Hebda et al. [113] reported PU hybrid foam (PUF), which was obtained by introducing with 0 to 15 wt % POSS chemically. The POSS moieties act as both pendant groups and cross-links. The incorporation of POSS leads to reduced porosity and increased hardness. The simulated physiological fluid (SBF) confirmed the material is bioactivity as well as the POSS used. Huang et al. [114] synthesized a series of hybrid PUs containing double-decker octaphenylsilsesquioxanetetraol (DDT_8_OH) and polyols through a one-step method. DMA analysis suggested that the enhanced *T*_g_ was due to the presence of B-POSS in the main chain. SEM images showed the presence of nano- and micro-sized B-POSS aggregates because of the heterogeneous dispersion of B-POSS in liner PU, which was further confirmed by the presence of a nanocrystalline phase of B-POSS by XRD analysis. The hydrophobicity and mechanical performance of the liner PU with B-POSS was obviously enhanced as well. Xu et al. [115] also investigated organic–inorganic polyurethanes with B-POSS, and found that the microphase separation of POSS was self-organized into spherical microdomains 10–50 nm in diameter. Raftopoulos et al. [116] reported the molecular dynamics and morphology of a polyurethane system with POSS through SEM, DSC, (thermally stimulated depolarization currents) (TSDC), and DMA. Different loadings of POSS in polyurethane resulted in different morphologies. The particles in the main chain had no influence on the time scale of segmental dynamics. The increase of *T*_g_ had no influence on the relaxation of α′. The incorporation of POSS in the polyurethane structure affected the glass transition, the crystallinity of the soft phase, and the order–disorder transitions [117].

### 3.2. Polyimide POSS

**Figure 13:**
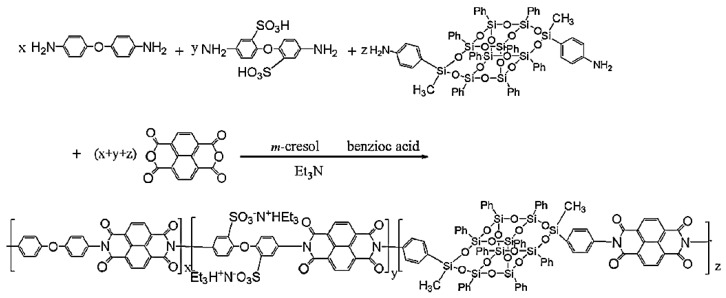
Synthesis of POSS-containing sulfonated polyimide in the main chain. (Reproduced with permission from Wu et al. [130]. Copyright 2015 Elsevier).

Polyimides are high-performance materials that display many advanced properties, such as for instance good resistance at high temperature, low water absorption, and alkali resistance. Thus, they have been widely used in many fields such as microelectronics and the aerospace industry [118]. Many efforts have been made to improve the thermal and mechanical properties of polyimides containing POSS [101,119,120,121,122,123,124,125,126,127,128,129]. Wu et al. [130] synthesized a novel polyimide with B-POSS in the main chains (Figure 13). Different POSS content in the main chain was realized via a multi-step reaction methodology to obtain a series of linear semiaromatic sulfonated polyimides. The TGA data showed that the weight loss of linear sulfonated polyimide POSS (SPI-x-POSS) (x: mole ratio (%) of phenylbisaniline-POSS) membranes was greatly increased by 200 to 450 °C. In addition, linear SPI-POSS copolymers displayed appreciable mechanical strength, good oxidative and hydrolytic stability, low methanol permeability, and high proton conductivity. Linear SPI-POSS-based copolymers were good potential candidates to fabricate proton exchange membrane (PEM) materials.

Liu et al. [68] synthesized a well-defined 3,13-dianilino B-POSS through the Heck reaction. The organic–inorganic polyimides prepared with 3,13-dianilino B-POSS displayed enhanced surface hydrophobicity compared to plain polyimide. The contact angle tested by water was increased by 20° with 19.4 wt % B-POSS, whereas plain polyimide was 87°. Dielectric measurement showed that dielectric constants were significantly lower and decreased with the increased content of compound **4**.

## 4. Conclusions

This review focuses on the functional methods of bifunctional POSS and composites with B-POSS in the main chain. B-POSS has great prospects for the development of large numbers of groups in organic–inorganic hybrid copolymers with B-POSS as the main component. Therefore, a number of breakthrough studies were done on the structures and properties of hybrid materials with B-POSS. Nanocomposites with excellent properties were constructed with the incorporation of POSS into linear polymers. Good thermal stability and mechanical properties and exceptional dielectric properties and solubility were reported through this modification. Moreover, in the future, POSS-containing monomers will be applied in various fields that require outstanding properties of polymers. Without a doubt, many exciting developments await POSS-containing hybrid materials, with the possibility for exciting new discoveries in the future.
